# Enhanced read resolution in reconfigurable memristive synapses for Spiking Neural Networks

**DOI:** 10.1038/s41598-024-58947-2

**Published:** 2024-04-17

**Authors:** Hritom Das, Catherine Schuman, Nishith N. Chakraborty, Garrett S. Rose

**Affiliations:** Department of Electrical Engineering and Computer Science, University of Tennessee, Knoxville, TN 37996 USA

**Keywords:** Current-controlled, Low power, READ current resolution, Read failure, Spiking Neural Network, Electrical and electronic engineering, Electronic devices

## Abstract

The synapse is a key element circuit in any memristor-based neuromorphic computing system. A memristor is a two-terminal analog memory device. Memristive synapses suffer from various challenges including high voltage, *SET* or *RESET* failure, and *READ* margin issues that can degrade the distinguishability of stored weights. Enhancing *READ* resolution is very important to improving the reliability of memristive synapses. Usually, the *READ* resolution is very small for a memristive synapse with a 4-bit data precision. This work considers a step-by-step analysis to enhance the *READ* current resolution or the read current difference between two resistance levels for a current-controlled memristor-based synapse. An empirical model is used to characterize the ${\hbox {HfO}}_{2}$ based memristive device. $1\textrm{st}$ and $2\textrm{nd}$ stage device of our proposed synapse design can be scaled to enhance the *READ* current margin up to $\sim$ 4.3$\times$ and $\sim$ 21%, respectively. Moreover, *READ* current resolution can be enhanced with run-time adaptation techniques such as *READ* voltage scaling and body biasing. The *READ* voltage scaling and body biasing can improve the *READ* current resolution by about 46% and 15%, respectively. TENNLab’s neuromorphic computing framework is leveraged to evaluate the effect of *READ* current resolution on classification, control, and reservoir computing applications. Higher *READ* current resolution shows better accuracy than lower resolution even when facing different levels of read noise.

## Introduction

The development of Artificial Neural Networks (ANNs) and Deep Neural Networks (DNNs) is inspired by the remarkable information processing abilities of mammalian brains, while also achieving low power consumption and minimal latency. Due to their exceptional classification accuracy, DNNs are attracting considerable interest as the preferred classifier in numerous machine learning and computer vision applications^1^. However, DNNs are typically executed on Von Neumann machines and are hence limited by the separation of memory and processing units, also known as the von Neumann bottleneck^2^. Besides that, the extensive computational requirements, high power consumption, and memory bandwidth associated with DNNs make them less attractive for mobile applications, where limitations in area and power are significant constraints^3^.

In order to address the limitations in power and memory capacities of traditional computing architectures mentioned above, coupled with further inspiration drawn from the efficiency of the biological nervous system, a novel concept of neuromorphic architectures has emerged. These architectures typically employ Spiking Neural Networks (SNNs), which aim to mimic the intricacies of the biological nervous system with greater fidelity by employing binary pulses as a means of communication. These architectures represent a distinct paradigm from the conventional Von Neumann architecture in terms of the co-location of memory and processing unit, demonstrating promising results in specific application domains^4–6^. Neuromorphic architectures not only offer better energy efficiency, but also promise parallel signal processing, fault tolerance, and reconfigurability. Furthermore, they can be implemented using diverse silicon-based technologies, large-scale architectures, and computational models of neural components^7–10^.

Neuromorphic systems, also referred to as Neuroprocessors, leverage the co-location of memory and processing units, where neurons act as the computational units, interconnected by synaptic memory elements. Synapses contain the weighted connections between neurons and can be implemented using digital^11,12^ or analog^13–19^ circuits. Nevertheless, incorporating a multitude of synapses presents several obstacles, including the efficient handling of storage space needed for weighted connections and the accommodation of diverse synaptic learning techniques that demand adaptable weight storage^9,20^. Memristors are potential candidates to address these issues. Memristors have proven to be more compact and power efficient for synaptic implementation compared to SRAM^21^ and capacitor-based implementations of the same resolutions^9,22^. Memristive synapses have also been shown to have extended memory retention time^22–24^.

First postulated by Leon Chua, the memristor is described as the fourth fundamental passive circuit element^25^. A memristor is a two-terminal device with analog memory properties that originate from its ability to switch resistance levels. When a voltage beyond a certain threshold is applied to its terminals, the resistance is modified. A memristor resistance state is also non-volatile making it a promising candidate for weight storage. Due to their compatibility with CMOS technology and non-volatile properties, memristors are well-suited for analog computation^18,26^.

In this work, we use a current-controlled synapse designed using a TiN TE (TE = Top Electrode)/${\hbox {HfO}}_{2}$/TiN BE(BE = Bottom Electrode) memristor^27^. To use this synapse, several operations need to be performed on the memristor, such as *FORMING*, *RESET*, *SET*, and *READ*. This memristor can vary its resistance in a range of a few k$\Omega$ to over 150 k$\Omega$^13,14^. However, for our design, we exclusively use the low resistance states (LRS) of the memristors due to improved reliability in this operating region, and to avoid a high degree of variability encountered near the high resistance states (HRS)^28^. The memristor can be programmed into different low-resistance states by precisely controlling the compliance current during the *SET* operation that overcomes the issues of variability and limited resolution^29^.

Although the synapse is designed while taking the reliability concerns into consideration, the synapse is vulnerable to limited resolution due to the use of a narrow resistance range in the low-resistance regions^30^. This occurs when the current generated by the different resistance states is not easily differentiable. This limitation can cause a reduction in the learning performance of SNNs^9,30^. Overlapping synaptic currents also make the synapse susceptible to noise and process variation^9^. Another disadvantage of the small difference between synaptic states is that it complicates the analog-to-digital converter (ADC) design significantly^9^. For the ADC to recognize different resistance states for digital conversion, the current output difference between the resistance states needs to be high enough for a compact, yet power efficient design^9^. This work aims at improving the current resolution of the memristive synaptic circuit using several techniques.

The key contributions of this paper are as follows.

1. *READ* current resolution of a current compliance memristive synapse is enhanced,
2. *READ* current resolution is enhanced with proper device scaling,
3. *READ* current resolution is made re-configurable at run time with *READ* voltage scaling,
4. *READ* current resolution is adaptable at run time with body biasing, and
5. the TENNLab neuromorphic software framework^31^ is utilized to observe the effect of *READ* current resolution on SNNs. Higher *READ* current resolution illustrates better accuracy with a lower possibility of a read error.

The remainder of this paper is organized as follows. The following section briefly describes a Verilog-A model for the hafnium oxide-based memristor device, the synaptic circuit built from this memristor, and a description of its *READ* operation. The next section shows the proper device sizing to enhance the *READ* current resolution. After that, a section illustrates two techniques for improving *READ* current resolution re-configurable at run time. The next section will evaluate the design performance based on different test cases. The next section exhibits the effect of the *READ* current resolution or weight resolution on Spiking Neural Networks (SNNs). A detailed comparison with prior works is analyzed in the next section. Finally, the paper is concluded with prospective future work.

## Current-controlled memristive synapse

### ${\hbox {HfO}}_{2}$ based device modeling

A Verilog-A model is utilized to simulate the ${\hbox {HfO}}_{2}$ based memristive devices^32^. In this model, mathematical equations are derived based on the memristance state and the required time to switch the states between HRS to LRS or LRS to HRS. The I–V characteristics of this device are taken under consideration to derive the empirical model of the memristor. The threshold voltage and switching time are two sets of important measured parameters for this model. There are some fitting constants in the model, which are utilized to fit the device’s I–V characteristics to observe the simulation behavior as closely as measured data. The sigmoid window function is also considered to be incorporated with different patterns of switching time between HRS to LRS and vice versa. This Verilog-A model is also capable of detecting the *RESET* failure if the *RESET* voltage has crossed its functional window. All mathematical equations, I–V curves, measured, and simulation details are available in a prior work^32^.

**Figure 1:**
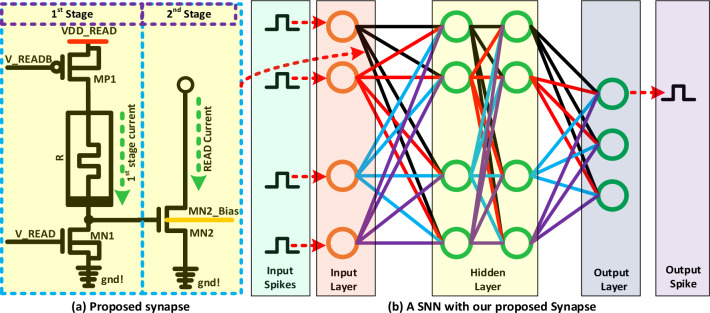
Memristor with *READ* circuitry is illustrated. (**a**) *READ* operation requires 1-PMOS and 2-NMOS. *READ* operation folded into two parts. $1\textrm{st}$ stage current is generated with $M_{P1}$ and $M_{N1}$. This current initiates a voltage to operate $M_{N2}$ in the linear region. Finally, the *READ* current will be sensed from the drain of $M_{N2}$. The body of the $M_{N2}$ is considered as a dedicated signal to control the threshold of this device. (**b**) A spiking neural network (SNN) is constructed with our proposed synaptic circuitry. Input spikes are fed into the input layer of neurons. Finally, the output spike indicates the class which is determined by the network.

### Proposed synapse

Memristors are widely used to construct brain-inspired synapses, which is the key element for neuromorphic computing. There are different synapse flavors based on the memristor’s recipe (combination of materials). Various materials are utilized to build the memristors such as ${\hbox {HfO}}_{2}$, ${\hbox {Ta}_{2}{\hbox {O}}_{5}}$, ${\hbox {NbO}_{2}}$, and so on. Our proposed architecture is designed using a TiN TE/$\hbox {HfO}_{2}$/TiN BE memristor. Figure 1a and b show the proposed synapse with *READ* devices and a simple SNN based on our synapse respectively. The first step of this synapse is the one-time *FORM* operation. Thick-oxide transistors are used for this design to take care of high voltage around 3.3 V for forming. A thick oxide transistor is also useful to reduce flicker noise. Unformed memristors usually exhibit resistance in the range of $\sim$ 8 to $\sim$ 10 M$\Omega$. After forming, the memristor’s resistance level will be in a few k$\Omega$. Hence, the synapse needs to *RESET* to a higher resistance state (HRS), which is typically hundreds of k$\Omega$. Finally, our device is ready to *SET* /write /program to a specific low resistance state from an HRS. Due to less variability, the programming region is selected in the LRS region. The targeted LRS for this design is from 5 to 20 k$\Omega$. Here, the LRS region is considered to program the synapse with a low inherent variation of ${\hbox {HfO}}_{2}$-based memristor. At the same time, we are sacrificing the low-power operation by eliminating the HRS region for programming. Here, we are targeting 4-bit precision with $\sim$ 1 k$\Omega$ resistance resolution. This design needs a set of *RESET* and *SET* to program in a new LRS value. After a successful *SET* operation, the synapse is ready for a *READ* operation.

Figure 1a shows the proposed current-controlled synapse with *READ* circuitry. $V\_READ$ and $V\_READB$ signals are utilized to access the memristor (R) during a *READ* operation. $VDD\_READ$ and $V\_READ$ are 1.2 V and 0.6 V respectively during a *READ* operation. In addition, 0 V is provided to the the $V\_READB$ node to access the memristor for a read operation. Due to the *READ* signal assertion, there will be a small $1\textrm{st}$ stage current through the memristor. This current will create a voltage to operate transistor $M_{N2}$. Finally, a *READ* *Current* is sensed from the drain of the $M_{N2}$. The body of this *MOSFET* is utilized as a separated signal to control the threshold voltage of $M_{N2}$. *READ* is a very sensitive operation for memristor-based synapses. Especially, the *READ* margin between two resistance levels (e.g. 5 k$\Omega$ and 6 k$\Omega$) needs to be good enough to read the data or weight properly. Most of the time the difference between the two resistance levels is a few nA for low-power design, which is very hard to sense properly. A research paper shows the *READ* current between 5 and 6 k$\Omega$ is 20 nA^28^. A few techniques can be utilized to overcome this low *READ* margin /resolution between two resistance levels. All the techniques are explained below with proper analysis.

## READ current resolution enhancement with proper device scaling

**Figure 2:**
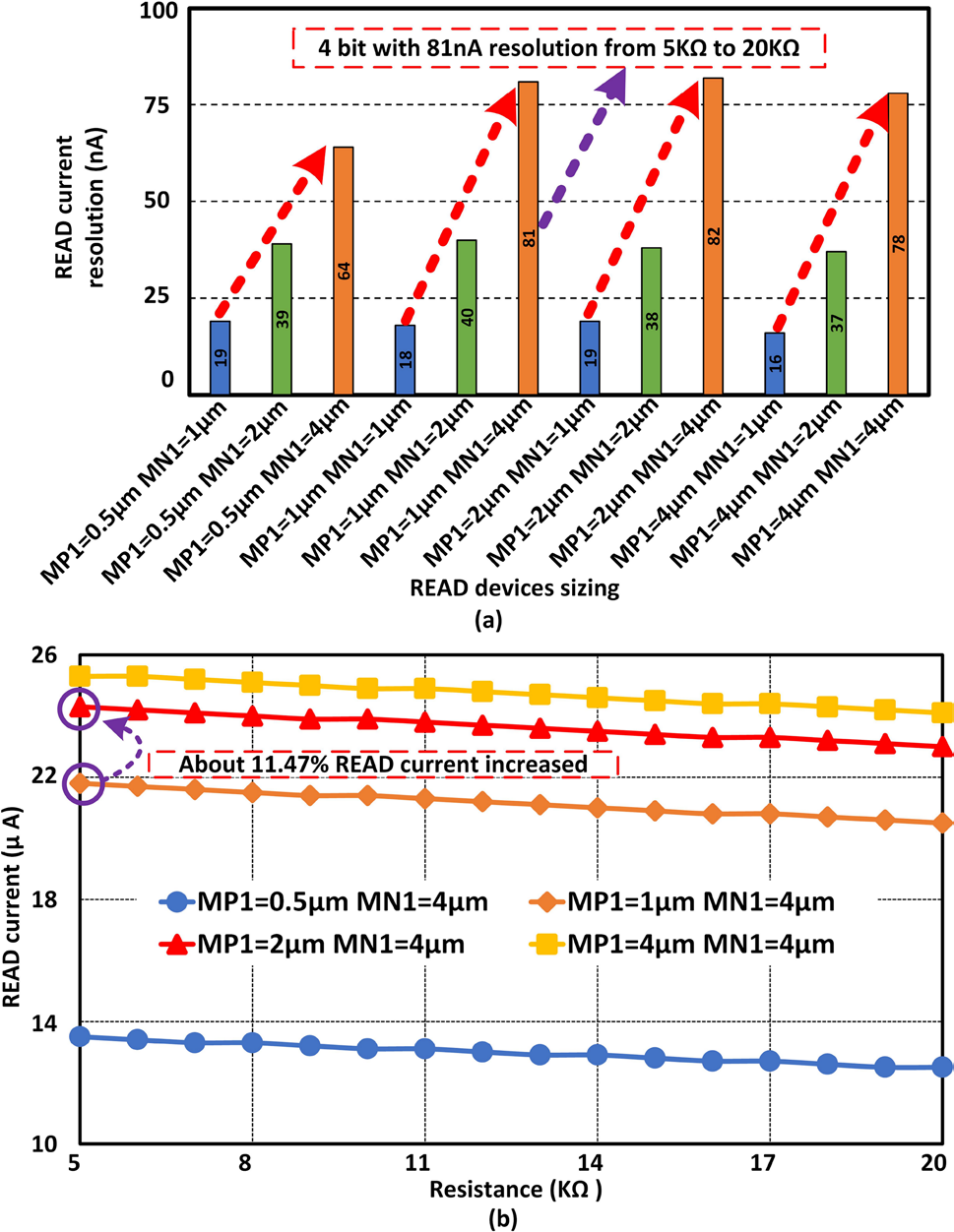
*READ* simulation results are illustrated based on the sizing of $M_{P1}$ and $M_{N1}$. The length and width of $M_{N2}$ are fixed at 0.5 $\upmu$m. (**a**) $M_{P1}$ is varied from 0.5 to 4 $\upmu$m. In addition, $M_{N1}$ is varied from 1 to 4 $\upmu$m. Larger $M_{N1}$ shows a higher impact on the *READ* current resolution. (**b**) Shows the *READ* current scale with different size of $M_{P1}$, when the width of the $M_{N1}$ is fixed at 4 $\upmu$m.

At first, *READ* device sizing is considered to observe the effect on *READ* current resolution. A 65 nm 10LPe CMOS process from IBM is utilized to construct and conduct Cadence Spectre simulations. A Verilog-A model is utilized to characterize the ${\hbox {HfO}}_{2}$ based memristor device^32^. Here, 9% of memristive variation is considered for the simulation, which is based on the testing results of memristive devices^28^. Figure 2a shows effect on *READ* current resolution with the sizing of $M_{P1}$ and $M_{N1}$. Here, the width and length of $M_{N2}$ are set at 0.5 $\upmu$m. In addition, the width of the $M_{P1}$ is varied from 0.5 to 4 $\upmu$m and the width of the $M_{N1}$ is varied from 1 to 4 $\upmu$m. In addition, the length of $M_{P1}$ and $M_{N1}$ (both are thick oxide transistor) are set at 0.5$\upmu$m. When the width of both $M_{N1}$ and $M_{P1}$ is minimal, the *READ* current resolution (one memristive level to another, e.g. 5–6 k$\Omega$) is at least 19 nA. Due to an optimized read procedure, the *READ* current shows very stable resolutions compared to prior work^28^. In this work, a regular pfet is utilized to control the read voltage at the drain of $M_N2$. Whereas a diode-connected pfet was connected in prior work. Hence, the width of the $M_{P1}$ set at 0.5 $\upmu$m and the width of the $M_{N1}$ varies from 2 to 4 $\upmu$m. The *READ* current resolution is 64 nA, when the width of the $M_{N1}$ and $M_{P1}$ are 4 $\upmu$m and 0.5 $\upmu$m respectively. The larger size of $M_{N1}$ and smaller size of $M_{P1}$ allow suitable gate voltage for the $M_{N2}$ to provide a high-resolution *READ* current. Twelve different sizing combinations are observed for *READ* current resolution. When the width of the $M_{P1}$ and $M_{N1}$ are 1$\upmu$m and 4 $\upmu$m, the *READ* current resolution is about 81 nA for 4-bit data precision. According to the last test case, if the width of $M_{P1}$ increases significantly and its size becomes the same as $M_{N1}$, then the *READ* current resolution does not show significant benefit on sizing. A better sizing combination is observed, when the $M_{N1}$ and $M_{P1}$ are not the same and $M_{P1}$ is smaller than $M_{N1}$. According to our sizing analysis, the *READ* resolution provides best performance when the width of $M_{N1}$ and $M_{P1}$ are 4 $\upmu$m and 1 $\upmu$m respectively. According to Fig. 2b, there is about 11.47% *READ* current overhead to improve only 1 nA current resolution. Next, the effect of length and width of the $M_{N2}$ is observed, with the width of the $M_{N1}$ and $M_{P1}$ transistors are set at 4 $\upmu$m and 1 $\upmu$m respectively.

**Figure 3:**
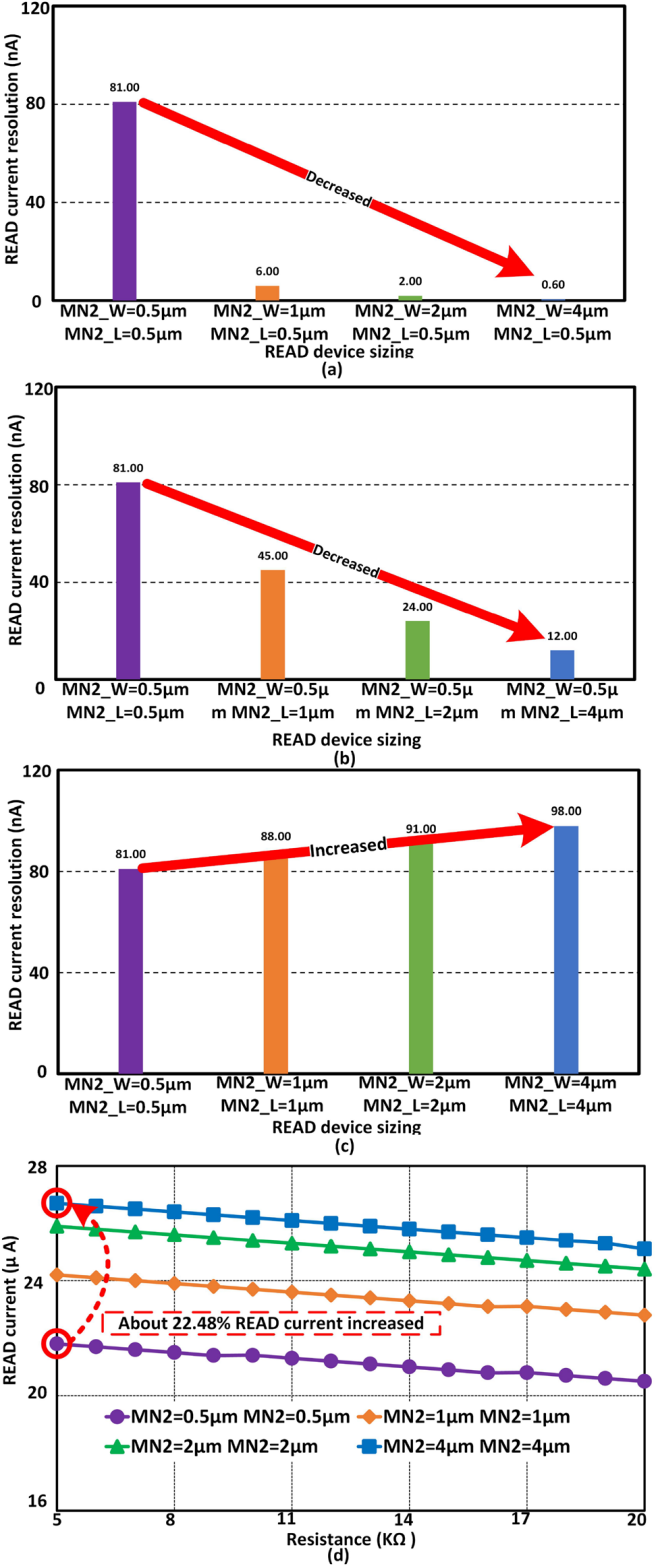
Cadence simulation results for *READ* current resolutions with different width and length of $M_{N2}$ device. (**a**) Shows the *READ* current resolution when the length of the $M_{N2}$ is fixed at 0.5 $\upmu$m and the width is varied from 0.5 to 4 $\upmu$m. The current resolution is drastically decreased with the increment of $M_{N2}$’s width. (**b**) shows the *READ* current resolution when the width of the $M_{N2}$ is fixed at 0.5 $\upmu$m and the length is varied from 0.5 to 4 $\upmu$m. The *READ* current resolution is also decreased with the increment of length of $M_{N2}$. Finally (**c**) Shows the *READ* current resolution when the length and width of $M_{N2}$ change simultaneously. *READ* current resolution is increased when the length and width of the $M_{N2}$ are increased at the same time. (**d**) Shows the *READ* current level with different $M_{N2}$ sizing. About 22.48% *READ* current overhead is observed to improve 21% READ current resolution.

The length and width of $M_{N2}$ are varied to observe the effect on *READ* current resolution. Figure 3a illustrates the *READ* current resolutions, when the width of the device is varied from 0.5 to 4 $\upmu$m and the length is fixed at 0.5 $\upmu$m. When the length and width of the device are set at a minimal size, the *READ* current resolution is about 81 nA. The first test case shows better *READ* current resolutions with lower power consumption.

Next, the width of the $M_{N2}$ is set at 0.5 $\upmu$m and the length is varied from 0.5 to 4 $\upmu$m. According to Fig. 3b, the *READ* current resolution is decreased with the increment of length. At this point, the overall *READ* current will be decreased by up-sizing the length of $M_{N2}$, with higher latency. Finally, both length and width are increased simultaneously. Figure 3c shows the *READ* current resolutions when both length and width are increased. *READ* current resolutions are increased as the length and width are up-scaled at the same time. About 21% improvement in *READ* current resolutions can be achieved by up-sizing the length and width of $M_{N2}$ simultaneously. Figure 3d shows the *READ* current level with different sizing combinations. *READ* current resolution can be increased with the overhead of area and *READ* current. Figure 3d shows, at 5 k$\Omega$ memristive weight, the *READ* current is 21.8 $\upmu$A with minimal length and width of the $M_{N2}$. On the other hand, when both length and width of $M_{N2}$ are increased to 4 $\upmu$m, the *READ* current is increased by 22.48%. At the same time, the *READ* current resolution is increased by 21%. There is a clear trade-off between *READ* current resolution and *READ* current overhead. In addition, the overall design area is also influenced by a larger length of $M_N2$.

Table 1 shows the optimized sizing configuration to enhance the *READ* current resolution. The width of $M_{P1}$ and $M_{N1}$ are set at 1 $\upmu$m and 4 $\upmu$m respectively. Both *MOSFET*’s length are fixed at 0.5 $\upmu$m. In addition, the length and width of $M_{N2}$ is considered as 4 $\upmu$m. The *READ* current resolution is about 98 nA with this optimized sizing. In the next section, the *READ* current resolution will be adapted dynamically with V_READ and MN2_Bias signals.

**Table 1:** Transistor scaling to enhance *READ* current resolution.

Transistor name	Width ($\upmu$m)	Length ($\upmu$m)
MP1	1	0.5
MN1	4	0.5
MN2	4	4

**Figure 4:**
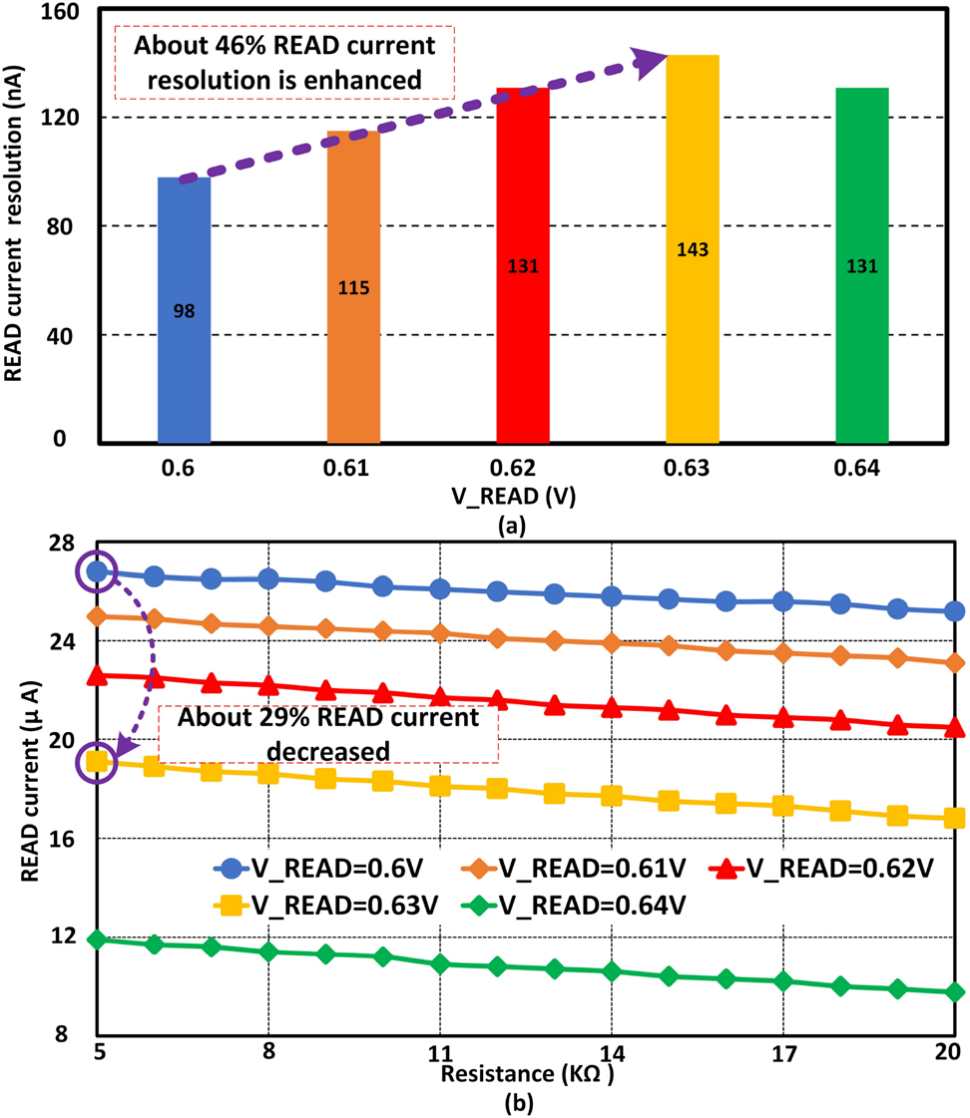
*READ* voltage (V_READ) has a significant effect on *READ* current resolution. (**a**) Shows the *READ* current resolution at different V_READ voltage. *READ* current resolution is increased with the increment of the gate voltage of $M_{N1}$. After a certain level of gate voltage increment, the *READ* resolution starts decreasing. (**b**) Exhibits the *READ* current level at different *READ* voltages. As we increase the *READ* voltage at the gate of $M_{N1}$, the *READ* current level starts decreasing. Due to a weak turn-on of $M_{N2}$, the *READ* current level is decreased. Here is an interesting thing to notice, as we increase the gate voltage of $M_{N1}$ the overall *READ* current level is decreased. As a result, the *READ* current resolution is increased with overall *READ* current optimization.

## Reconfigurable READ current resolution

*READ* current resolution can be adapted at run time. Various applications can perform better at enhanced *READ* current or weight resolution. To enhance the application’s performance, a reconfigurable or run-time adaptation of *READ* current resolution is proposed with different circuit techniques. Specifically, *READ* voltage (V_READ) scaling is a useful technique to influence the *READ* current resolution at run time.

### READ voltage scaling

Figure 1 shows V_READ is the gate voltage of $M_{N1}$. Initially, the applied amplitude of this signal was 0.6 V. Figure 4a shows the *READ* current resolution at 0.6V is 98 nA for 4-bit data. Here, the optimized sizing is utilized from Table 1. The *READ* current resolution is about 143 nA at 0.63 V as V_READ. About 46% of *READ* current resolution can be enhanced with V_READ scaling at run time. If the V_READ is increased more than 0.63 V for this particular sizing or configuration, then the resolution is decreased. A stronger turn-on of $M_{N1}$ influences the final *READ* current negatively. As a result, the resolution is decreased with excessive V_READ. Figure 4b shows *READ* current with different gate voltages of $M_{N1}$. When the *READ* voltage is 0.6 V, the *READ* current is about 26.8 $\upmu$A at 5 k$\Omega$. On the other hand, if the *READ* voltage is scaled up to 0.63 V, the *READ* current will be decreased to about 19.1 $\upmu$A. About 29% *READ* current can be optimized when the *READ* voltage is scaled from 0.6 to 0.63 V. *READ* current shows a significantly lower value at 0.64 V. However, at 0.64 V the *READ* current resolution is reduced significantly compared to the value at 0.63 V. In addition, at 0.64 V the std. dev. of *READ* current is $\sim$ 7% higher than std. dev. at 0.63 V. Thus, the *READ* current at 0.63 V is more reliable than at 0.64 V. According to our design optimization, the *READ* current resolution is higher at 0.63 V among all the *READ* voltages. In addition, the *READ* current resolution can be varied from 98 to 143 nA with V_READ signal scaling. In the next sub-section, another device technique is utilized to enhance the *READ* current resolution at run time.

**Figure 5:**
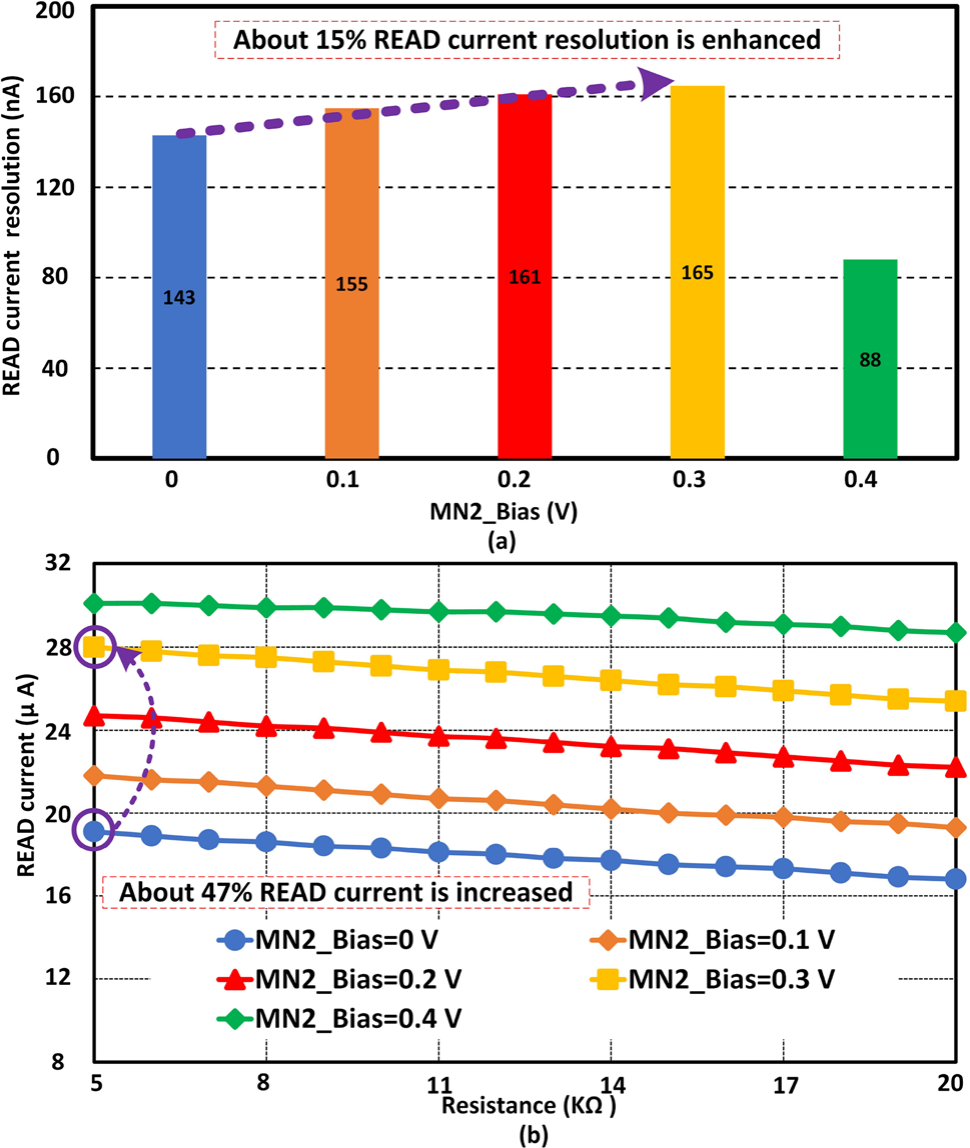
*READ* device $M_{N2}$ plays an important role to make *READ* current resolution adaptable at run time. The body of $M_{N2}$ is scaled to enhance the resolution with *READ* power overhead. (**a**) Shows the body biasing effect on *READ* current resolution. About 15% resolution can be enhanced with body biasing. (**b**) Illustrates the *READ* current level at different biasing voltages. The *READ* current is increased with the increment of body biasing of $M_{N2}$.

### READ device $M_{N2}$ biasing

*READ* device $M_{N2}$ biasing is another technique to manage synaptic *READ* current at run time. Here, the *READ* voltage V_READ is set at 0.63V with optimized sizing. Figure 5a shows the *READ* current resolution at different bias voltages at the body of $M_{N2}$. As we know, body biasing can change the threshold voltage of a *MOSFET* to control the current flowing through it. Here, a positive body voltage is applied on $M_{N2}$ to improve the *READ* current resolution. When the body voltage is 0 V, the *READ* current resolution of our proposed synapse is about 143 nA. If the body voltage is scaled up to 0.3 V, then the *READ* current resolution is further up to 15%. In addition, if the body biasing voltage is increased more, then a reverse phenomenon is observed with *READ* current resolution. Due to the channel effect of the NMOS, the reverse phenomenon is observed with higher body bias voltage. At 0.4 V the *READ* current resolution is decreased to 88 nA. As a result 0.3 V is an optimized body bias voltage for this design scenario. Figure 5b exhibits the *READ* current of our synapse at different body biasing scenarios. Overall *READ* current is increased with the increment of body biasing voltage. About 47% *READ* current is increased with body biasing to enhance the *READ* current resolution at run time. Finally, it can be said that the *READ* current resolution of our proposed synapse can be adapted at run time with different body biasing voltages.

## READ current resolution and design performance evaluation

Table 2 shows an evaluation of device sizing, *READ* current resolution, and *READ* power. There are five test cases considered for the evaluation. The first test case is constructed with base device sizing. Here, the V_READ and body bias (MN2_Bias) are 0.6 V and 0 V respectively for first three test cases. The first test case shows 19 nA *READ* current resolution with 18.87 $\upmu$W as a max *READ* power. Here, both stages’ power ($1\textrm{st}$ and $2\textrm{nd}$) are considered for the max *READ* power. The second test case is the $1\textrm{st}$ stage device sizing. Here, the *READ* current resolution is 81 nA with 4-bit precision which is about 4.3$\times$ higher than the base sizing test case. Only 5% of power overhead is observed compared to the base test case. The next test case is focused on $2\textrm{nd}$ stage device sizing with $1\textrm{st}$ stage sizing. Here, the *READ* current resolution is 98 nA, which is 5.16$\times$ more improved than the base test case (Base sizing). Here, the *READ* power improvement is only 0.16% compared to the base test case. When the $2\textrm{nd}$ stage device is scaled the overall *READ* current is optimized slightly compared to the base test case. The fourth test case is to increase the V_READ to 0.63 V from 0.6 V. In this scenario, the *READ* current resolution is 143 nA, which is 7.53$\times$ enhanced than the base test case. In addition, the max *READ* power shows 1.43% overhead compared to the base test case. Finally, the body biasing is applied to the body of $M_{N2}$. After applying 0.3 V, the *READ* current resolution is about 165 nA, which is 8.68x improved compared to the base test case. Here, the max *READ* power overhead is about 3.60% compared to the base test case. The *READ* voltage on the source of the $M_{N2}$ is automatically adjusted based on the gate voltage of the $M_{N2}$. Due to that, the *READ* power is quite stable in this design. Here, the Monte Carlo simulation is observed with 1000 samples in a Cadence Spectre environment to analyze the *READ* current variation. The *READ* current shows about 0.65$\times$ variations with 8.68$\times$ resolution improvement.

**Table 2:** *READ* current resolution enhancement and design evaluation.

Test case	Device size	READ current resolution	READ current resolution evaluation	Max READ power [both stage power]	Max READ power evaluation
Base sizing	MP1 = (0.5/0.5) $\upmu$m MN1 = (1/0.5) $\upmu$m MN2 = (0.5/0.5) $\upmu$m	19 nA	–	18.87 $\upmu$W	–
1st stage device sizing	MP1 = (1/0.5) $\upmu$m MN1 = (4/0.5) $\upmu$m MN2 = (0.5/0.5) $\upmu$m	81 nA	4.26$\times$ improved compared to base sizing	19.82 $\upmu$W	5% overhead compared to base sizing
2nd stage device sizing	MP1 = (1/0.5) $\upmu$m MN1 = (4/0.5) $\upmu$m MN2 = (4/4) $\upmu$m	98 nA	5.16$\times$ improved compared to base sizing	18.84 $\upmu$W	0.16% improved compared to base sizing
V_READ @0.63V	MP1 = (1/0.5) $\upmu$m MN1 = (4/0.5) $\upmu$m MN2 = (4/4) $\upmu$m	143 nA	7.53$\times$ improved compared to base sizing	19.6 $\upmu$W	1.43% overhead compared to base sizing
Body bias @0.3 V	MP1 = (1/0.5) $\upmu$m MN1 = (4/0.5) $\upmu$m MN2 = (4/4) $\upmu$m	165 nA	8.68$\times$ improved compared to base sizing	18.19 $\upmu$W	3.60% improved compared to base sizing

**Figure 6:**
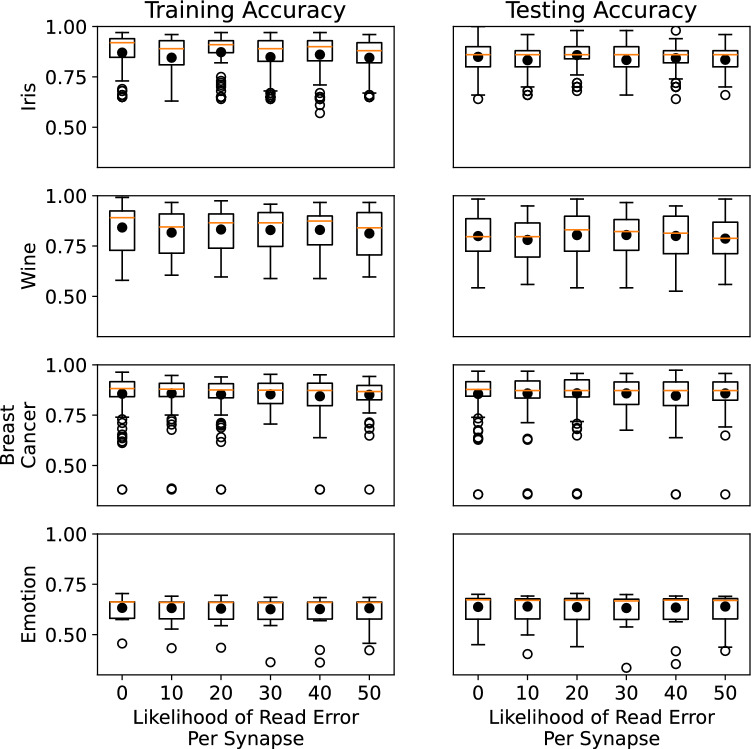
Training and testing accuracy for iris, wine, and breast cancer datasets. In these results, the networks were trained and tested with read errors.

**Figure 7:**
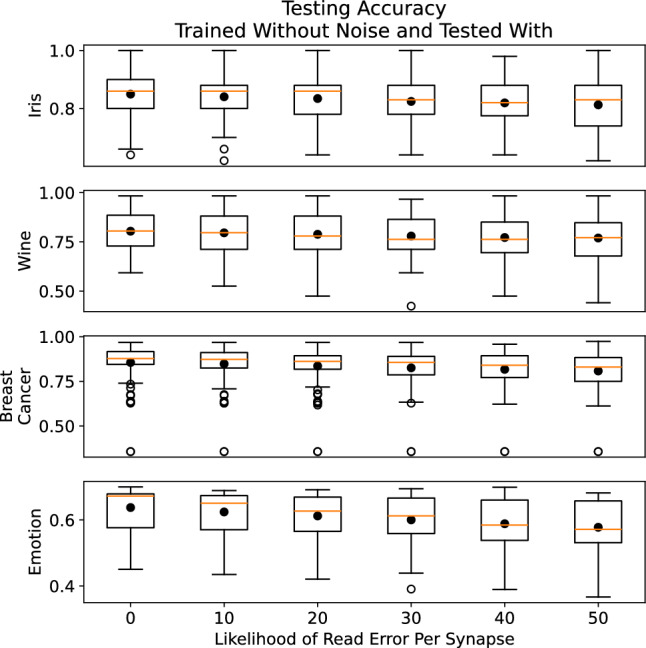
Testing accuracies for networks trained without read errors and tested with read errors.

**Figure 8:**
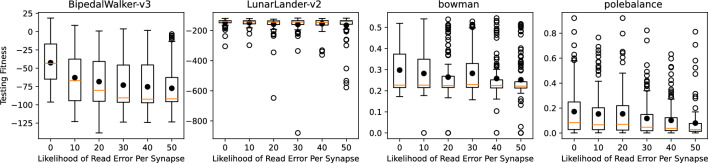
Results for control with read errors on synapses.

**Figure 9:**
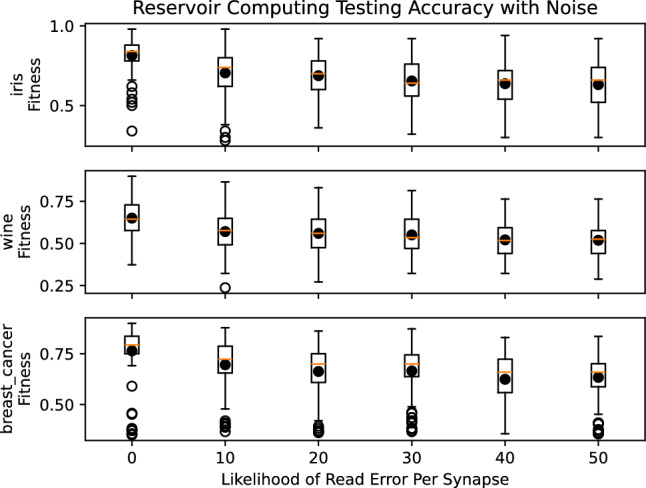
Results for reservoir computing with read errors on synapses.

**Figure 10:**
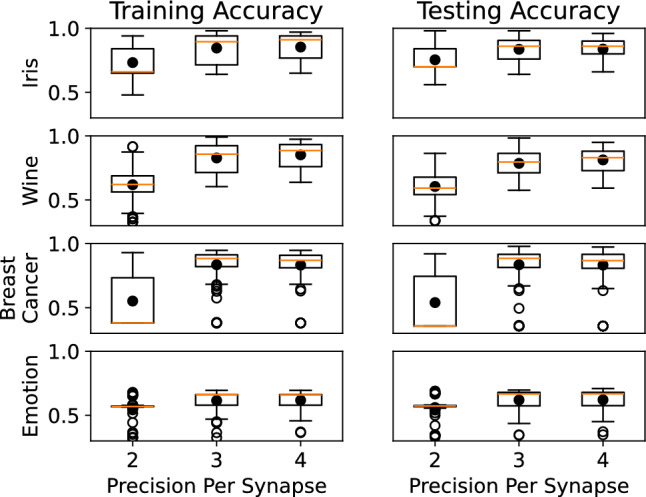
Results for reduced precision on the synaptic weights.

**Figure 11:**
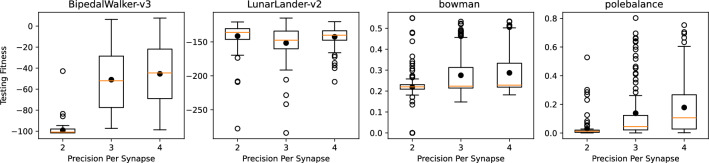
Results for control with reduced precision on the synaptic weights.

**Figure 12:**
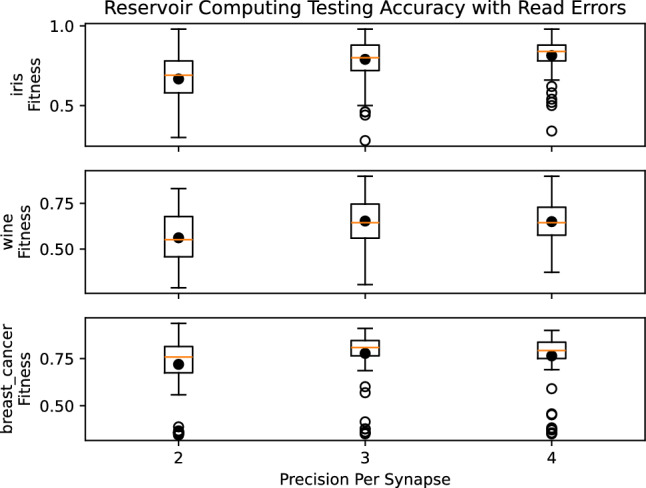
Results for reservoir computing with reduced precision on the synaptic weights.

## Impact of READ current resolution on applications

To specifically investigate the impact of READ current resolution, we assessed the performance of spiking neural networks under varying probabilities of synapse read failures. Our hypothesis is that when the current resolution is higher, there will be fewer or no read errors, while when the current resolution is lower, read errors are significantly more likely. To perform this evaluation, we leveraged the TENNLab neuromorphic computing framework ^31^, which allows for evaluation of neuromorphic processors using different applications and algorithms. Within the framework, we used the RISP neuromorphic simulator ^33^, with integrate and fire neurons and synapses with 4-bit weight resolution.

To specifically study the impact of READ current resolution, we evaluated how spiking neural networks with different likelihoods of read failures for each synapse. In particular, for a particular network evaluation, we defined a likelihood of read failures for each synapse read, wherein the weight read would be one level off (either the level above or level below), which would be more likely to happen for low current resolution. We trained the networks with these read failures using an evolutionary optimization training approach for spiking neural networks and neuromorphic system called EONS ^34^. EONS evolves the parameters and topology of the network simultaneously. We trained for the iris dataset, the wine dataset, and the breast cancer dataset, three commonly used toy datasets in machine learning that are available in the UCI machine learning repository ^35^, as well as the EEG motion dataset, a timeseries dataset ^36,37^. We trained 100 networks for each of six different likelihoods of read errors for the synaptic weight values: 0, 10, 20, 30, 40, and 50 percent for each dataset. Figure 6 shows the results for these simple datasets on both training and testing performance. This figure shows that, in general, the best overall testing performance was achieved by no read errors at all; however, on average, some noise on the read errors does not necessarily hurt performance significantly, either in training or testing. For the results in Fig. 6, it is worth noting that the networks were trained and tested using read errors. Figure 7 shows the results for when networks are trained without read errors and then tested with varying likelihoods of read errors per synapse, which would likely be the case for networks trained in simulation and then deployed to hardware. In this case, we can see that read errors cause a decrease in testing performance for each dataset.

We conducted similar tests for four test applications: two from the OpenAI gym control environments (BipedalWalker-v3 and LunarLander-v2) ^38^ and two from the TENNlab suite of control applications (bowman and polebalance) ^39^. The results for different likelihoods of read errors for these control tasks are shown in Fig. 8. In this case, we do see a significant downward trend in performance for most of the applications when training and testing with noise.

Finally, in Fig. 9, we see the results of a reservoir computing approach when encountering read errors on synapses. We use reservoirs of 100 neurons with 10% randomly initialized connectivity. We once again evaluate the three toy classification datasets, but we omit the timeseries EEG dataset. In this case, we see a significant downward trend, more pronounced and more consistent than in the other cases. As such, this indicates that the reservoir approach may not handle noise well.

Because this work enables higher-precision weights to be used on synapses with more reliability, we investigated the impact of precision on performance. Figure 10 shows the results for different levels of bit-precision (2, 3, and 4) on the synaptic weights. Here, we can see that 2-bit weight synapses perform significantly worse across all three datasets than 3- and 4-bit precision synapses, as expected. We see similar results for the control tasks (Fig. 11) and the reservoir computing tasks (Fig. 12).

**Table 3:** Comparison with prior works.

References	JETCAS,2023^28^	MWSCAS,2023^30^	Electron Device Lett.,2010^40^	Appl. Phys.,2017^41^	Semicond. Sci., 2015^42^	RSC. Adv.,2016^43^	Nanoscale, 2014^44^	Phys. Status Solidi A, 2017^45^	This work
Technology	65 nm CMOS	65 nm CMOS	–	–	–	–	–	–	65 nm CMOS
Memristor material	${\hbox {HfO}}_{2}$	${\hbox {HfO}}_{2}$	$\hbox {TiO}_{2}$	$\hbox {TiO}_{2}$	${\hbox {HfO}}_{2}$	a-ZnO	${\hbox {HfO}}_{2}$	$\hbox {TiO}_{2}$	${\hbox {HfO}}_{2}$
Programming region	5 k$\Omega$–20 k$\Omega$	3 k$\Omega$– 18 k$\Omega$	1 k$\Omega$–1 G$\Omega$	10 k$\Omega$–100 M$\Omega$	10 k$\Omega$– 1 M$\Omega$	24 M$\Omega$–176 M$\Omega$	0.8 k$\Omega$–100 M$\Omega$	1 M$\Omega$– 10 G$\Omega$	5 k$\Omega$–20 k$\Omega$
Impact of process variation	Lower	Lower	Higher	Higher	Higher	Higher	Higher	Higher	Lower
Number of programming states	16	16	4	6	4	5	8	6	16
Storage density improvement compared to prior work	1$\times$	1$\times$	4$\times$	2.67$\times$	4$\times$	3.2$\times$	2$\times$	2.67$\times$	1$\times$(base)
Max READ current	$\sim$5.44$\upmu$A	$\sim$2.92$\upmu$A	$\sim$90$\upmu$A	100$\upmu$A	10$\upmu$A	4.166 nA	$\sim$625$\upmu$A	0.5$\upmu$A	28$\upmu$A
Max READ power	$\sim$8.24$\upmu$W	$\sim$3.50$\upmu$W	$\sim$45$\upmu$W	100$\upmu$W	1$\upmu$W	0.4116 nW	$\sim$312.5$\upmu$W	0.25$\upmu$W	19.6$\upmu$W
Max READ power improvement compared to prior work	No	No	Yes	Yes	No	No	Yes	No	–
Minimum READ current resolution	20 nA	20 nA	90 nA	$\sim$17 nA	81 nA	0.462 nA	45 nA	0.45 nA	165 nA
READ current resolution improvement compared to prior work	8.25$\times$	8.25$\times$	1.83$\times$	$\sim$9.7$\times$	2.04$\times$	357$\times$	3.67$\times$	366.67$\times$	1$\times$
READ current linearity	Linear	Linear	Non-linear	Non-linear	Non-linear	Non-linear	Non-linear	Non-linear	Linear

## Comparison with prior works

Reliable *READ* current resolution is a big challenge for the memristive-based synapse or memory design. Usually, the minimum current resolution is a few nA. Due to that, it is challenging for the circuit designer to sense the current level properly with ADC or CMOS neurons. In our neural network analysis, we consider a CMOS neuron to observe the charge accumulation and fire^46^. In this work, a memristor-based synapse is optimized to enhance the *READ* current resolutions. At first, the device sizing is considered to optimize the resolution. Hence, *READ* voltage and body bias are considered to enhance the resolution at run time. Here, the optimal size is adopted from Table 1. In addition, the *READ* voltage and body bias voltages are selected to 0.63 V and 0.3 V from 0.6 and 0 V respectively. According to Table 3, the minimum *READ* current resolution is 165 nA with 4-bit data precision. As we know, the larger resistance level shows a lower current resolution. In our case, the current level difference between 19 and 20 k$\Omega$ is considered to determine the minimum current resolution. At room temperature the value is at least 165 nA. The maximum *READ* current $\sim$ 28 $\upmu$A is captured at 5 k$\Omega$. Our synapse is programmed at LRS to avoid inherent process variations.

Another research article shows the minimum *READ* current resolution of a 3T1R synapse is 20 nA for 4-bit data precision. Our proposed design shows 8.25$\times$ resolution improvement compared to their work^28^. Energy-efficient and high-performance synapse is presented in another research paper, where the minimum *READ* current resolution is 20 nA^30^. This design is also based on ${\hbox {HfO}}_{2}$ based memristor and 65 nm CMOS process. The programming region is between 3 to 18 k$\Omega$, which provides low inherent process variation with 4-bit data density. Our proposed design also illustrated 8.25$\times$ current resolution improvement compared to their work^30^. A $\hbox {TiO}_{2}$ based memristive memory is presented in^40^, which is programmed from 1 k$\Omega$ to 1 G$\Omega$. Due to the utilization of HRS, the inherent process variation is higher for this design compared to our proposed design. This design only covers four programming states. As a result, our proposed design shows a 4$\times$ more dense data storage capacity compared to their design^40^. The max *READ* current is about 3.21$\times$ higher than our proposed design. The minimum *READ* current resolution is about 90 nA, which is 1.83$\times$ lower than our proposed design.

A research group is presented a $\hbox {TiO}_{2}$ based multi-level resistive memory, which is programmed from 10 k$\Omega$ to 100 M$\Omega$^41^. Due to programming in HRS, the inherent process variation is higher than our design. Their design is only programmed in six different states, which exhibits 2.67$\times$ lower memory density compared to our proposed design. The maximum *READ* current of their design is 100 $\upmu$A, which is 3.57$\times$ higher than our proposed design. Moreover, the minimum *READ* current resolution of their design is $\sim$ 17 nA, whereas our proposed design shows $\sim$ 9.7$\times$ enhanced resolution compared to their design^41^. A ${\hbox {HfO}}_{2}$ based multi-level cell is presented with 2-bit memory density, which is 4$\times$ lower memory density than our proposed synapse^42^. Their device is programmed from 10 k$\Omega$ to 1 M$\Omega$, which shows higher inherent process variation compared to our design. Their device draws lower maximum *READ* current than our design. However, the minimum *READ* current resolution is 2.04$\times$ lower than our proposed design.

Another multi-level resistive memory is presented using a-ZnO material, which is programmed between 24 and 176 M$\Omega$^43^. Their design shows higher process variation and 3.2$\times$ lower memory density than our proposed design. Their maximum *READ* current is lower than our design. However, the minimum *READ* current resolution is extremely low compared to our proposed design. A ${\hbox {HfO}}_{2}$ based multi-level RRAM is presented in a research article, where authors programmed their device between 0.8 k$\Omega$ and 100 M$\Omega$ with 8 programming states^44^. Our proposed design shows lower process variation and 2$\times$ higher memory density than their design. The maximum *READ* current of their design is extremely higher than our proposed design. Our proposed design also shows a 3.67$\times$ improved minimum *READ* current resolution than their design^44^. Another research group presented their device with $\hbox {TiO}_{2}$, which is programmed between 1 M$\Omega$ and 10 G$\Omega$ with six programming states^45^. This design shows a 2.67$\times$ lower memory density than our proposed design. Due to their HRS programming region, the process variation is higher than our design. The maximum *READ* current of their design is lower than our proposed design. However, their minimum *READ* current resolution is extremely lower than our proposed design. Moreover, our design shows power savings compare to prior works^40,41,44^. In addition, our proposed design shows more linear *READ* current compared to other designs^40–45^. Due to a compact programming range, our design shows better linearity compared to prior works. A wide programming range causes non-linear behavior of *READ* current.

After comparing our proposed design with prior works from different research groups, it can be said that our proposed design shows lower inherent process variation with higher memory density. In addition, our proposed design shows an enhanced *READ* current resolution compared to others’ designs.

## Conclusions and future work

In this paper, a ${\hbox {HfO}}_{2}$ based current-controlled memristive synapse is optimized for *READ* operation. At first, the *READ* devices are optimized to enhance the *READ* current resolution. About 4.3$\times$ and 21% READ current resolution is enhanced with $1\textrm{st}$ and $2\textrm{nd}$ stage device sizing respectively. *READ* voltage scaling and body biasing are applied to enhance the *READ* current resolution at run time. About 46% and 15% *READ* current resolution is improved with *READ* voltage scaling and body biasing. A neuromorphic framework EONS shows that a higher *READ* current resolution exhibits better accuracy compared to a lower resolution on classification and control applications. Lower resolutions are more likely to be affected by reading failures with higher noise. As a result, a higher *READ* current resolution makes the neuromorphic system more reliable.

## Data Availability

The datasets (circuit simulation) generated during this study are available from the corresponding author (H.D.) upon reasonable request.

## Code availability

The codes for application evaluation are available from the author (C.S.) upon reasonable request.
