# Author Correction: Enhanced read resolution in reconfigurable memristive synapses for Spiking Neural Networks

**DOI:** 10.1038/s41598-024-62798-2

**Published:** 2024-05-23

**Authors:** Hritom Das, Catherine Schuman, Nishith N. Chakraborty, Garrett S. Rose

**Affiliations:** Department of Electrical Engineering and Computer Science, University of Tennessee, Knoxville, TN 37996 USA

Correction to: *Scientific Reports* 10.1038/s41598-024-58947-2, published online 17 April 2024

The Keywords section in the original version of this Article was incorrect.

“Current-controlled, Low power, Stochastic computing, Approximate computing, Spiking Neural Network"

now reads:

“Current-controlled, Low power, READ current resolution, Read failure, Spiking Neural Network”

The original Article has been corrected.

